# A Rapid Evidence Assessment on The Effectiveness of Interventions for Autistic Adolescents with Harmful Sexual Behaviors

**DOI:** 10.1177/15248380241241024

**Published:** 2024-03-29

**Authors:** Sebastian Trew, Douglas Hugh Russell

**Affiliations:** 1 Australian Catholic University, Watson, ACT, Australia

**Keywords:** sexual abuse, child abuse, intervention, sexual assault, prevention of child abuse, treatment/intervention, adolescents, sexual harassment

## Abstract

The management and treatment of harmful sexual behaviors (HSBs) in autistic adolescents is a complex area of research and clinical practice. Autistic adolescents face unique challenges in understanding social and sexual interactions, putting them at a higher risk of engaging in HSBs. Existing research on interventions for HSBs among autistic adults is growing, but evidence for adolescents is not well understood. Thus, understanding the effectiveness of interventions targeting HSBs in autistic adolescents is crucial. We conducted a rapid evidence assessment to review peer-reviewed research on the effectiveness of interventions for autistic adolescents at risk of or engaging in HSBs. In all, 12 studies met the criteria for review. Inclusion criteria required articles to be published in a peer-reviewed journal, be related to HSB prevention and intervention in adolescents aged 12 to 18 with autism spectrum disorder, be written in English, and include original data. Six databases were used, and we screened the titles and abstracts of 34 studies. The reviewed studies described cognitive-behavioral therapy, pharmacological interventions, family involvement, and multidisciplinary team approaches in addressing HSBs. However, the literature has significant limitations and we suggest that the literature is not robust enough to indicate a promising evidence-based approach for interventions for autistic adolescents who are at risk of or who display and engage in HSBs, and the findings are not transferable to practice. Additional research is required to better prepare healthcare professionals for addressing HSBs in autistic adolescents.

## Introduction

Autism spectrum disorder (ASD) or “autism” is a neurodevelopmental disorder characterized by deficits in social communication and interaction, and the presence of restricted and repetitive patterns of behavior, interests, or activities (American Psychiatric Association, 2022). The traits of autism include difficulties with social communication, which manifests as difficulty reading social cues, perspective-taking, and verbal communication. There is also a preference for routine, sameness, and predictability. Difficulties in these areas and the interaction between these traits and the environment may impact an individual’s mental health, for example, through social isolation and increased anxiety (Acker et al., 2018; Factor, 2019; Mattys, 2018; Sibeoni et al., 2022; Spain et al., 2017). The impact of autism also varies depending on an individual’s stage of development, environmental demands, and the presence of other conditions such as learning disabilities, depression, attention deficit hyperactivity disorder, or anxiety. In addition, the effects of autism may change over time (National Health Service, 2020).

In our rapid evidence assessment (REA), we use identity-first language to describe autistic people. Recent empirical studies have shown that autistic individuals tend to prefer to identify first language and/or the neutral term “person on the autism spectrum” (Bottema-Beutel et al., 2020; Bury et al., 2020; Kenny et al., 2016). We use the term “disorder” when referring to the diagnosis of ASD and use neutral terms such as “condition” and “disability” when referring to individuals.

## Background

### Autism and Harmful Sexual Behaviors

Harmful sexual behaviors (HSBs) have been increasingly recognized as areas of concern (Dredge & Rose, 2022; McLay et al., 2015; Maggio et al., 2022; Sevlever et al., 2013) in autistic adolescents and adults.

HSBs—also referred to in the literature as problematic sexual behavior or inappropriate sexual behavior—involve engaging in sexual activities that are potentially harmful, risky, or otherwise inappropriate for the individual or others involved (Hackett, 2014). These behaviors can have negative consequences for physical, emotional, and mental well-being, and they can also cause distress for the person engaging in them, as well as for others who may be affected (Hackett et al., 2019). Evidence in the literature across different populations highlights how, as with sexual abuse perpetrated by adults, the use and display of HSBs by children and adolescents can be traumatic to both others and themselves (Hailes et al., 2019; Wamser-Nanney & Campbell, 2020). These behaviors lead to poor outcomes in the domains of physical and psychological health, neurobiological and development, interpersonal relationships, connection to community and culture, and sexual identity (Cashmore & Shackel, 2013; Nagtegaal & Boonmann, 2021; Royal Commission into Institutional Responses to Child Sexual Abuse, 2017).

Autistic children and adolescents are no more likely to engage in problematic or HSBs than typically developing children and teenagers (Allely & Creaby-Attwood, 2016; Sevlever et al., 2013; Weiss & Fardella, 2018). A study by Visser et al. (2017) found no difference in the ability to judge the appropriateness of different sexual situations between “cognitively able” autistic adolescents and typically developing peers. However, young people with diagnoses of intellectual disability and/or ASC are over-represented among juvenile sex offenders (Schnitzer et al., 2020). Other than these studies, there is a dearth of literature supporting an understanding of the prevalence of HSBs in autistic adolescents or the contexts in which it might be more prevalent. The management of HSBs in autistic adolescents is of great importance, as it can lead to negative consequences for the individual, their family, the community, and the broader. HSBs can significantly impact the lives of autistic individuals, their families, and their caregivers, leading to increased social isolation, disrupted family life, and exclusion from the community (including incarceration), educational, and vocational opportunities (Murrie et al., 2002).

### Factors Contributing to HSBs in Autistic Adolescents

Autistic adolescents who display HSBs may face unique challenges when it comes to understanding and navigating social and sexual interactions (Clionsky & N’Zi, 2019). Due to difficulties with social communication, understanding social cues, and comprehending complex social situations, they may be at a higher risk of engaging in or displaying HSBs (Allely & Creaby-Attwood, 2016; Sevlever et al., 2013). In addition, social factors, such as inappropriate social modeling and lack of social boundaries, may also play a role in the development of HSBs in autistic individuals (Weiss & Fardella, 2018). Due to the complex nature of HSBs and ASCs, specialized interventions are likely needed to address this issue effectively.

Potential factors of problematic and HSBs among children and adolescents include difficulties in social skills and a lack of understanding of social conventions, sensory issues, the inability to distinguish public from private spaces, difficulties understanding others’ thoughts, social gestures, language, and emotions, or the ramifications of their behavior (Dredge & Rose, 2022; Haskins & Silva, 2006; Murrie et al., 2002; Payne et al., 2020). Other reasons why this group might engage in problematic or harmful sexual behavior might be due to restricted interests, which may include atypical sexual interests (Payne et al., 2020). A brief overview of the factors that may contribute to the risk of HSBs among autistic adolescents is briefly summarized below.

#### Social Skill Deficits

Autistic adolescents may struggle with understanding social cues, reading body language, and interpreting the feelings and intentions of others. This can lead to misinterpretations or misunderstandings in social and sexual situations, potentially resulting in inappropriate behaviors.

#### Limited Understanding of Social Norms

Autistic adolescents may have difficulty grasping social norms and expectations, which could lead to inappropriate sexual behaviors that are unintentional or stem from a lack of understanding.

#### Lack of Sexual Education

Autistic adolescents may not receive adequate sex education that is tailored to their specific needs and learning styles. This can result in a lack of knowledge about appropriate sexual behavior, consent, and boundaries.

#### Restricted Interests and Repetitive Behaviors

Some autistic adolescents may develop an intense interest in a specific topic, including sex or sexual material. This interest may lead to inappropriate or HSBs if not addressed and managed appropriately.

Given the above, autistic adolescents with HSBs require specialized interventions that consider the individual’s unique cognitive, social, and emotional needs, as well as the complex interplay of psychological and psychosocial factors that contribute to this group displaying and engaging in HSBs.

### Evidence Base for Addressing HSBs Among Autistic Adolescents

Several programs and interventions are designed to address and prevent HSBs among non-autistic young people (Campbell et al., 2020; Meiksans et al., 2017; Quadara et al., 2020). These programs can be educational, therapeutic, or community-based and often focus on promoting healthy relationships, improving communication skills, and fostering self-awareness. Treatment and support for non-autistic individuals who engage in HSBs may involve therapy, support groups, or medical intervention, depending on the specific circumstances and the severity of the behavior.

Interventions specific to treating a range of inappropriate, problematic, harmful, or abusive sexual behaviors in autistic people over 18 years of age include cognitive-behavioral therapy (CBT), pharmacological interventions, and behavioral interventions (Dredge & Rose, 2022; McLay et al., 2015; Maggio et al., 2022; Sevlever et al., 2013). However, the efficacy of these interventions is not well established, and more research is needed to determine the most effective interventions for autistic adults.

Although there is a growing body of research on interventions for non-autistic young people addressing HSBs, and for autistic adults addressing HSBs, the evidence bases specifically addressing HSBs among autistic adolescents is limited. Existing reviews relating to sexual abuse and autistic adolescents are often focused on preventing abuse of autistic adolescents (Farlina & Allenidekania, 2019; Govindshenoy & Spencer, 2007; Stobbe et al., 2021; Wissink et al., 2015) as opposed to reviewing evidence of how to prevent autistic adolescents from displaying HSBs. One review (Sala et al., 2019) investigated relationships and sexuality education in autistic individuals with an intellectual disability and found that most programs focused on keeping participants safe from experiencing abuse themselves and to a lesser extent the anatomy and mechanics of sexual relationships. None of these reviews focused on autistic individuals who display HSBs as adolescents or are sexually abusive in adulthood themselves. Some studies have shown the effectiveness of interventions in improving social skills, emotion regulation, and communication, which may indirectly contribute to reducing the risk of HSBs. However, direct evidence for the impact or effectiveness of interventions targeting HSBs in this population is scarce. Some intervention approaches that have shown promise for individuals with ASC in general include:

#### Social Skills Training

Social skills training programs have been demonstrated to improve social functioning in autistic adolescents. Although these programs may not directly target HSBs, they can help individuals with ASC better navigate social situations, which could indirectly reduce the risk of inappropriate sexual behaviors.

#### Cognitive-Behavioral Therapy

CBT is effective in addressing various mental health concerns in autistic individuals, such as anxiety and depression. Adapting CBT to focus on HSBs may be a promising approach, but further research is needed to establish its effectiveness for this specific issue.

#### Psychoeducation and Sex Education

Providing tailored sex education for autistic individuals can help them better understand appropriate sexual behaviors, consent, and boundaries. Although there is some evidence to suggest that adapted sex education can be beneficial for individuals with ASC, more research is needed to determine its effectiveness in specifically reducing HSBs.

#### Family Therapy and Parent Training

Family therapy and parent training have been shown to improve family functioning and communication, which may indirectly reduce the risk of HSBs. However, research on the direct impact of these interventions on reducing HSBs in autistic adolescents is limited.

Overall, there is a need to better understand the effectiveness or impact of interventions specifically targeting HSBs in autistic adolescents and to develop and evaluate approaches for this group. Although some intervention approaches have shown promise in addressing related issues, it is of high priority to investigate within the peer-reviewed literature the interventions that address the unique challenges and needs of this cohort. Furthermore, we consider that those who display HSBs in adolescence can potentially go on to use abusive behavior as adults. Therefore, we think it is important to understand which interventions best prevent the display of HSB in adolescents.

## Question

This REA was assigned to understand what peer-reviewed research has discovered about the effectiveness or impact of interventions for autistic adolescents who are at risk of or who display and engage in HSBs. The question this REA seeks to address is, *what is known in the peer-reviewed scientific literature about the effectiveness of interventions for autistic adolescents with HSBs?* The aim of this REA is to provide recommendations on the next steps in the development of research and interventions for this cohort.

## Methodology

### Inclusion Criteria

We used the Center for Evidence-Based Management Guideline for REAs (Barends et al., 2017) to conduct this REA. We included articles that were published in a peer-reviewed journal and were related to the prevention and intervention/treatment of HSBs in autistic adolescents between the ages of 12 to 18. The articles had to be written in English and include original data. The research had to involve an intervention or program aimed at reducing engagement in HSBs or the risk of engaging in such HSBs. The risk factors as identified and suggested in the literature included difficulties in social skills and a lack of understanding of social conventions, sensory issues, the inability to distinguish public from private spaces, difficulties understanding others’ thoughts, social gestures, language, and emotions, or the ramifications of their behavior, and restricted sexual interests. Outcome variables had to include either a display of or a measure of engagement in HSBs or related risk factors of engaging in HSBs. The studies could be of any type or design, as long as they meet the above criteria.

This REA is guided by the definition of HSBs developed by Hackett et al., 2019 (derived from Hackett, 2014), that is, “. . .Sexual behaviours expressed by children and young people under the age of 18 years old that are developmentally inappropriate, may be harmful toward self or others, or be abusive toward another child, young person or adult” (p. 13).

### Search Strategy and Process

The following six databases were used to identify studies: Medline, PsycInfo, ERIC, CINAHL, Web of Science (All fields search), and Scopus. No limiters were applied to the search strategy. The search conducted used combinations of terms such as, adolescen*, “harmful sexual* behavio*,” “autism spectrum diagnos*” OR ASD OR autis*. We conducted seven different search queries and screened the titles and abstracts of 34 studies. The PICOC table (Population Intervention Comparison Outcome Context) and an outline of search terms and queries are provided in Appendix A. The search process described above is in Appendix B and shows the concepts, the search terms used, how the terms were combined, and the number of studies found in each database and includes the date that the search was performed, and the search filters applied during the search.

### Study Selection

**Figure 1.**
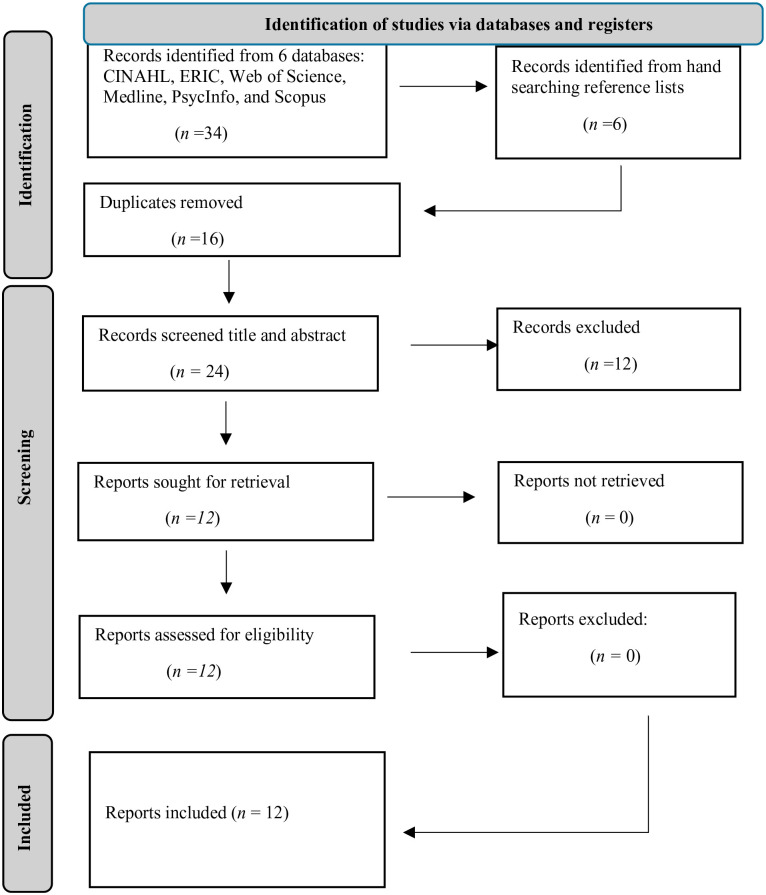
Selection process chart. *From*: Page et al. (2021).

The studies retrieved from the search process (Figure 1) were screened by the first author for inclusion criteria. In addition, the reference lists of studies retrieved were screened to identify articles for possible inclusion in the REA. Screening was undertaken as a two-stage process. In stage 1, the titles and abstracts of the studies retrieved through the searches were reviewed by the first author. Each abstract was compared against the inclusion criteria, and if met, was set aside for a full study review in stage 2. If any doubt was had, the study was included. At the end of this process, a total of six articles were identified as eligible for stage 2 full study review.

The reference lists of these six articles were then hand-searched, with six more articles being identified. The title and abstracts of these six articles were screened and from this process, all were set aside for a full study review in stage two. At the end of this process, a total of 12 articles were identified as eligible for a full study review in stage 2.

In stage two, author one read each of the 12 articles included from stage one in full and compared them against the inclusion criteria. To ensure the trustworthiness of the REA findings, author two took a random sample from the studies included and independently reviewed them for inclusion criteria. To quantify the degree of agreement with the first author, the inter-rater reliability (IRR) was assessed (Hallgren, 2012). Disagreements were solved by discussion and consensus between reviewers. The IRR’s magnitude indicates that the extent of agreement among the reviewers is good, IRR is 1 (or 100%). In all, 12 records were included in our REA.

The selection process described above is provided below. This shows the articles obtained from each database, articles obtained from search, duplicates removed, titles and abstracts screened for relevance, studies excluded, studies full text screened for relevance, and a final number of included studies.

### Data Extraction

A standardized data extraction template was utilized for each included study, following recommendations from the Cochrane Handbook for Systematic Reviews of Interventions (Higgins et al., 2022). A summary of the extracted data is found in Table 1.

**Table 1.** Data Extraction.

Authors and Year	Participants and Characteristics	Behavior	Study Design	Intervention	Intervention Administers	Outcomes	Key Findings
Shenk and Brown (2007)	14-year-old white male, borderline cognitive functioning, history of sexual offenses	Sexual deviancy, prior sexual offenses, excessive/public masturbation, deviant fantasies	AB design, case study, quantitative, cognitive-behavioral therapy (CBT) approach	Daily group therapy, weekly individual CBT, 45-week residential program, exposure and response prevention	Cognitive-behavioral therapist in a residential treatment facility	Reduced arousal, masturbation, and recidivism risk; improved emotional communication and empathy	Successful treatment and reintegration into family and community
Thompson and Beail (2002)	18-year-old male, with severe intellectual disabilities, auto-erotic asphyxiation	Daily auto-erotic asphyxiation	AB design, qualitative case study, staged intervention procedure	Desensitizing to the penis, teaching appropriate masturbation techniques, home practice with monitoring	Multidisciplinary team (general practitioner, psychologists, other health professionals) in outpatient setting and patient’s home	Reduction of the dangerousness of the behaviors, slow progress in generalization	Behavioral intervention did not eliminate auto-erotic asphyxiation
Pritchard et al. (2016)	17-year-old white British male, autism spectrum disorder (ASD), harmful sexual behavior	Sexual abuse history and convictions of sexual offenses including sexual touching, gestures, comments, threats, aggression, absconding, disruption, self-harm	AB design, quantitative, multi-component behavioral intervention	Points and level system, active support, CBT, sex and relationship education, offense-specific intervention	Trained staff at a residential special school	Reduced harmful/inappropriate sexual behavior, increased community visits	Benefits of intensive, long-term multi-component treatment in residential settings
Visser et al. (2017)	189 adolescents (12–18 years) with ASD	Adolescents with self-reported problematic sexual behaviors to receive psychosexual development, interpersonal boundaries, and communication skills education	Randomized controlled trial, tackling teenage training (TTT) program	Psychoeducation and communicative skill practice related to puberty, sexuality, and intimate relationships	Professionals (bachelor’s/master’s in psychology/social services) with experience working with clients with an ASC.	Improved psychosexual knowledge, insight, social functioning, decreased problematic sexual behavior	TTT program effective for younger adolescents with ASD
Griffin-Shelley (2010)	A 14-year-old male with Asperger’s, sex addiction, and sexual offending behavior	Diagnosis of sex addiction and sexually offending behavior toward family members and others. Frequent public masturbation, and accessing pornography	Case study, CBT approach	Group therapy, psychoeducation, relapse prevention, individual therapy sessions	Therapists trained in a CBT approach.	Possible reduction in recidivism behaviors, continued lying, and deception	The addiction model of treatment may be more effective than the offender model, community aftercare showed positive changes
Ray et al. (2004)	Four male adolescents with autism spectrum condition. Case study 1: 15-year-old male with ASC. Case study 2: 17-year-old male with ASC. Case study 3: 16-year-old Caucasian male with ASC. Case study 4: 14-year-old African American male with ASC	CS1: Sexually inappropriate behaviors toward others including sexually threatening behaviors; CS2: paraphilic tendencies, masturbation using objects; CS3: sexually coercive and aggressive behaviors toward children; CS4 sexually inappropriate behavior and obsessive preoccupation with sex	Case studies	Residential multidisciplinary treatment package adapted for ASC, based on CBT, narrative elements, involving family/carers, and direct care staff	Therapists trained in CBT and direct care staff.	Anecdotal evidence of improvements in flexibility in thinking, emotional regulation skills, willingness to try new things, and psychosexual problems	Recommendations for interventions include tailoring to individual needs, involving family, training direct care staff, using evidence-based approaches, and continuous monitoring
Deepmala and Agrawal (2014)	A 13-year-old boy with severe autism	Hypersexual behavior and aggression	Narrative case report using a qualitative design	Low-dose propranolol (0.3 mg/kg/d, or 10 mg twice daily) prescribed at a psychiatric clinic	A psychiatrist at a psychiatric clinic.	Decreased hypersexual behavior in school and home settings, reduced pacing behavior	Propranolol therapy was effective in reducing hypersexual behaviors without reported side effects
Kohn et al. (1998)	16-year-old boy diagnosed with Asperger’s syndrome, conduct disorder, and IQ of 120	Aggressive and sexual offenses	Single case study with a narrative and qualitative design	Series of residential treatments focusing on pharmacotherapy (levopromazine, lithium, carbamazepine, propanol, and cyproterone acetate), psychotherapy, and family therapy	Team of professionals, including psychiatrists.	Anecdotal evidence of substantial improvement in harmful sexual behavior (HSB) with the introduction of a beta-blocker and antiandrogen	Hospital admissions alone were insufficient, but combined pharmacotherapy led to improved behavior
Fosdick and Mohiuddin (2016)	A 15-year-old male with ASD and intellectual disability	Severe sexual aggression toward young brother and other children	Single case study, narrative, and qualitative approach	Leuprolide acetate (25 mg every 3 months), behavioral therapy (3–4 times per week), monitored in a community setting	Psychiatrist and behavior analyst.	Anecdotal evidence of reduced sexual aggression during leuprolide acetate use; problematic behavior reoccurred during a gap in medication and resolved when resumed	Leuprolide acetate is effective in reducing sexual aggression with some side effects
Coskun and Mukaddes (2008)	13-year-old boy with ASD, moderate mental retardation, severely impaired language development, and stereotypic play patterns	Inappropriate sexual behavior (ISB), fetishist behavior, sexual aggression, and agitation	Case report using qualitative ABA design	Mirtazapine (15 mg/day) for 10 weeks	Psychiatrist.	Fetishistic behavior ceased during treatment and reemerged when stopped	Positive treatment effects, but limitations include lack of experimental design, unclear outcome measures, and no follow-up data
Coskun et al. (2009)	Eight male participants aged 12–16 years with ASD and various co-occurring conditions	Inappropriate sexual behavior (ISB) including masturbation, foot fetishism, touching and rubbing women, irritability, aggression, touching women’s breasts, disrobing, self-biting, and socks fetishism.	AB design with 8-week treatment	Mirtazapine with an initial dosage of 7.5–15 mg/day, increased to a maximum of 30 mg/day	Team of psychiatrists.	Improvement in excessive masturbation and other ISB; reduced time spent in ISB, with some participants almost stopping ISB entirely	Positive treatment effects, but limitations include lack of experimental design, no direct measures of change in ISB, and no follow-up data
Nguyen and Murphy (2001)	13-year-old white male with autism, no verbal communication	Excessive masturbation in home and public settings, and minimal appropriate social interaction	Case report using qualitative AB design	Mirtazapine, after an initial unsuccessful behavioral intervention	Psychiatrist in a clinical child mental health unit.	Improved frequency of nightly masturbation, cessation of public displays of masturbation, better sleep, increased hunger, improved social relatedness	Mirtazapine was effective in reducing excessive masturbation and improving social relatedness

## Results

In all, 12 papers were included in our REA. Six papers described a range of non-pharmacological interventions as the primary treatment, while six papers described a range of pharmacological interventions as the primary treatment of HSBs in autistic adolescents.

### Critical Appraisal of Studies

#### Methodological Appropriateness and Quality

It is crucial to ascertain the trustworthiness of scientific studies, considering their validity and reliability (Barends et al., 2017). We evaluated the methodological appropriateness and quality of the studies included in this REA by considering the six levels of appropriateness, derived from the classification systems proposed by Shadish et al. (2002) and Petticrew and Roberts (2008). To assess the methodological quality of the studies, we used Kmet et al.’s (2014) standard quality assessment criteria for evaluating primary research papers from a variety of fields.

After critical appraisal of the 12 included studies for this REA, the overall quality of the included studies was assessed to be low, with most studies rated at the lowest level for appropriateness (Petticrew & Roberts, 2008; Shadish et al., 2002) and poorest scoring for quality (Kmet et al., 2014). The items the studies scored low for on the quality assessment included evident and appropriate study design, method of subject/comparison group, sample size, use of verification measures to establish credibility and address researcher bias, outcome and exposure measures, analytic methods, and reporting of results.

In just one study (Visser et al., 2017), a randomized control trial scored highly. For instance, all but three of the studies included in this REA were single case study reports. As a result, the trustworthiness of the scientific evidence supporting the following main findings is limited.

### Study Characteristics

#### Country

The studies in this REA included participants from the following countries: U.S. (Deepmala & Agrawal, 2014; Fosdick & Mohiuddin, 2016; Griffin-Shelley, 2010; Nguyen & Murphy, 2001; Ray et al., 2004; Shenk & Brown, 2007); UK (Pritchard et al., 2016; Thompson & Beail, 2002); Turkey (Coskun & Mukaddes, 2008; Coskun et al., 2009); The Netherlands (Visser et al., 2017); and Israel (Kohn et al., 1998).

#### Setting

The studies in this REA described interventions administered within a range of settings, including residential treatment facilities, community settings, outpatient settings that is, transitioning to a patient’s home, special education schools, mental health institutions, and psychiatric clinical/medical settings.

#### Professionals Involved

The studies included in this REA involved various professionals to administer interventions and treatments for adolescents. These professionals included cognitive-behavioral therapists, multidisciplinary teams consisting of psychologists, general practitioners and health professionals, trained staff at residential special schools, professionals with bachelor’s or master’s degrees in psychology or social services experienced in working with participants with ASD, psychiatrists, and behavior analysts. These professionals included in the interventions administered them in diverse settings such as residential treatment facilities, outpatient settings, community settings, patient’s homes, special schools, and psychiatric clinics.

#### Participant Characteristics

The studies included 210 participants with varied characteristics, primarily focusing on male autistic adolescents (82%). Participant ages ranged from 12 to 18 years, with most studies focusing on individuals with various degrees of intellectual disabilities or cognitive impairments. The sample sizes were mostly single case studies (Coskun & Mukaddes, 2008; Deepmala & Agrawal 2014; Fosdick & Mohiuddin, 2016; Griffin-Shelley, 2010; Kohn et al. 1998; Nguyen & Murphy, 2001; Pritchard et al., 2016; Ray et al., 2004; Shenk & Brown, 2007; Thompson & Beail, 2002) and with one larger group (Visser et al., 2017), which included 189 participants (*n* = 152 males) aged 12 to 18.

Three single case studies (Nguyen & Murphy, 2001; Pritchard et al., 2016; Shenk & Brown, 2007) reported on the ethnicity of the participants (*n* = 3), as white and one other study (Ray et al., 2004) reported their participants as white (*n* = 1) and African American (*n* = 1). The remaining eight studies reported no ethnicity of the participants so the percentage of ethnicities among the sample is not known. Several studies reported on participants’ communication impairments as verbal or nonverbal or comorbid diagnoses including conduct disorder, major depression, and attention deficit hyperactivity disorder. Characteristics of the adolescents in the included studies are shown in Table 1.

The studies included in this REA encompass a wide range of HSBs displayed by autistic adolescents. Many of the behaviors for which the adolescents were receiving intervention or treatment were those directed at others (e.g., sexual assault and abuse of younger children, HSBs directed at younger children or peers, problematic sexual behavior with others such as family members including younger siblings, the sexual touching and rubbing of women, sexual touching of others to generate self-stimulation, hypersexual behaviors, sexual gestures and aggression, and sexually abusive and/or sexually offensive behavior).

Other behaviors for which adolescents were receiving intervention or treatment were self-inflicted or directed at the self and were reported to be either causing harm to the adolescent themselves or were observed to be problematic or inappropriate and as causing distress for others, including family members and those in the community (e.g., at school) or were behaviors that the adolescent engaged in or displayed in a public setting. These behaviors were reported to be excessive masturbation, fetishist behavior or sexually deviant behavior, auto-erotic asphyxiation, paraphilia, touching one’s genitals in public, disrobing, pinching nipples, and ejaculation in public. We found no mention by study authors about the proposed function of the HSBs displayed by the participants. Descriptions of HSBs displayed by adolescents are shown in Table 1.

#### Study Interventions

Most study authors reported on individualized interventions involving one participant, an autistic adolescent with HSBs, and often with identified comorbid diagnoses or reported behavioral or mental health issues.

Four of the included studies (Coskun & Mukaddes, 2008; Coskun et al., 2009; Deepmala & Agrawal, 2014; Nguyen & Murphy, 2001) utilized pharmacotherapy as the sole treatment of HSBs in autistic adolescents. Six of the included studies (Griffin-Shelley, 2010; Pritchard et al., 2016; Ray et al., 2004; Shenk & Brown, 2007; Thompson & Beail, 2002; Visser et al., 2017) utilized a range of non-pharmacological interventions to address HSBs in autistic adolescents. The remaining two of the included studies utilized pharmacotherapy (Fosdick & Mohiuddin, 2016; Kohn et al., 1998) alongside other therapeutic modalities.

The interventions and treatments in the studies utilized various strategies and methods to address the behaviors displayed by the adolescents. These included CBT, exposure and response prevention (ERP), psychoeducation, relapse prevention, pharmacotherapy, and behavioral interventions. Several interventions were identified to include a broader intervention outcome or treatment goal to address community safety, addressing harm caused, preventing further harm, promoting well-being, and education—described as psychoeducation related to puberty, sexuality, intimate relationships, sex and relationship education, and psychosexual education.

Key outcomes of several of the interventions included managing and reducing sexual arousal, challenging cognitive distortions, enhancing empathy, and developing relapse prevention plans. Some studies involved parental monitoring, structured residential programs, and tailored staff training. Interventions were often personalized and adjusted based on the adolescent’s individual needs, with some employing pharmacological treatments (including propranolol, mirtazapine, or leuprolide acetate), alongside behavioral therapy.

Just one study (Visser et al., 2017) was reported as focused on the primary preventive psychoeducational effects of a program (Tackling Teenage Training). The author’s rationale for a prevention focus to the study for investigating HSBs among autistic adolescents was due to ethical concerns about placing adolescents with obvious psychosexual issues on a waiting list for 1 year, as per study intervention requirements. In addition, the authors identified that there is an urgent need for further research to explore how an increase in psycho-sexual knowledge and insight into the self can improve romantic skills and prevent the development of problematic sexual behavior and victimization among this cohort. Intervention details are provided in Table 1.

#### Intervention Outcomes

Most articles reported on the success of the intervention for the treatment of adolescent’s HSBs. Authors reported that the intervention and treatment for HSBs among this cohort led to a reduction in both the behavior and the risk of the outcomes of the behavior (e.g., a reduction in arousal and masturbation and a reduction in recidivism), as well as success in the patient’s transition or integration back into the private and/or public setting.

In addition to a reported reduction in the problematic or harmful behavior, authors reported a series of improvements to other outcomes including, improved social responsiveness, higher psychosexual knowledge, increased flexibility in thinking (i.e., less rigidity in thinking pattern and style as associated with ASC), acknowledgment of the need to regulate urges, improved social relatedness, and better sleep.

For interventions including pharmacological therapy as either sole or in-part treatment for the HSBs of autistic adolescents, the behaviors re-emerged as per baseline at cessation of treatment but were reduced again once treatment was re-administered.

## Discussion

This REA aimed to identify and examine the effectiveness of interventions for addressing HSBs in autistic adolescents. The studies included in this review provided evidence for a range of interventions, including pharmacological treatments, CBT, and other psychotherapeutic approaches. The findings, however, are to be interpreted with caution as the overall quality of the included studies was assessed to be low, with most studies rated at the lowest level for appropriateness (Petticrew & Roberts, 2008; Shadish et al., 2002) and scored poorly in quality (Kmet et al., 2014). Just one study (Visser et al., 2017), a randomized control trial, scored highly.

Given this, we suggest that the literature is not robust enough to indicate a promising evidence-based approach for interventions for autistic adolescents who are at risk of or who display and engage in HSBs, and the findings are not transferable to practice. Additional research is required to better prepare healthcare professionals for addressing HSBs in autistic young people.

### Study Design and Methodological Issues

Although the case studies and single case designs reviewed in our REA provide anecdotal evidence of intervention effectiveness, future research should use rigorous research designs to establish evidence-based treatments for autistic adolescents with HSBs. A significant and key limitation in the current literature is the lack of experimental designs, including randomized controlled trials, which would provide more robust evidence for the efficacy of the interventions. Rigor is meticulous adherence to the scientific method to guarantee the strength and impartiality of experimental design, methods, analysis, interpretation, and result dissemination. It emphasizes the importance of complete openness in disclosing experimental procedures to enable replication and expansion of the findings by others (Hofseth, 2018). This would provide a more robust basis for clinical and educational decision-making and contribute to the development of effective and tailored interventions. In addition, many studies did not provide detailed information on the measures used to assess changes in HSBs, and there was a lack of inter-observer agreement, follow-up data, and generalization data among most studies.

Comprehensive and standardized assessment tools are needed to better understand the specific needs and challenges of this population, as well as to measure the effectiveness of interventions. The studies reviewed in our REA used a variety of measures to assess HSBs and treatment effectiveness. Future research should focus on developing more standardized and reliable measures of HSBs and treatment outcomes for use in clinical practice and research.

### Limited Representation of Diversity

From the small samples of adolescents in study interventions, most were recorded as being white and male. This finding highlights the paucity of knowledge on the topic and with autistic females who display HSBs. It also highlights the limited knowledge of diversity and differences, including cultural context, socioeconomic status, race and ethnicity, gender identity, and sexual orientation of autistic adolescents.

A consideration for future research is to ensure homogeneity among samples to be able to accurately suggest particular treatment options for autistic adolescents with varying degrees of social skills, intellectual capability, and emotional understanding. To do this, an understanding of the function of HSBs being displayed by participants is required, to support the theoretical “through line” from function (i.e., sensory seeking, social connection, or intense interest) to treatment approach (i.e., sensory integration, social interventions, or CBT for intense interests). Future research should consider interventions underpinned by specific functions as this would significantly impact the development of the intervention.

### Involvement of Families, Caregivers, and Care Staff in Treatment

Noteworthy, the studies suggest the importance of involving families, caregivers, and direct care staff in the treatment process. A collaborative, supportive and consistent environment is crucial for the effectiveness of interventions for autistic adolescents, as it ensures the continuity of care and fosters the generalization of skills to real-life situations (Trew, 2024). A recent systematic review of how child sexual abuse prevention programs engage parents has found parents are most often included by either communicating program details through workshops with them or incorporating learning at home activities to solidify outcomes (Russell et al., 2024).

Such concepts should be considered and evaluated in the context of involving families in the prevention of HSBs in autistic adolescents. Studies should aim to develop interventions that are effective in reducing HSBs and improving quality of life while also being feasible and acceptable for both the individuals and their caregivers. Training for direct care staff to better understand the motivations behind HSBs among this cohort and develop appropriate strategies to address them is also crucial.

### Identify Effective Pharmacological Treatments

Although several studies have examined the use of pharmacological treatments for HSBs, the literature in this area is still limited. Future research should investigate the efficacy of pharmacological treatments for autistic adolescents with HSBs, with a focus on identifying potential side effects and optimal dosing regimens and with consideration for the rights of autistic individuals.

### Examine the Effectiveness of Different Intervention Components

Different components (e.g., CBT, ERP, psychoeducation, pharmacological) in isolation and combination should be assessed, to determine the most effective treatment modalities for addressing HSBs in this group. Many of the studies reviewed in our REA utilized a multidisciplinary approach that included medication, behavioral therapy, and family therapy. Future research should continue to explore the effectiveness of individual and combined treatment modalities to develop comprehensive intervention packages for autistic adolescents with HSBs.

### Investigate Potential Moderators and Mediators

Treatment effectiveness may be affected by factors such as age, gender, cognitive ability, social skills, and comorbid mental health conditions. Research examining these factors can assist with a better understanding of the contributors to successful intervention outcomes.

### Examine the Long-term Outcomes

Although many studies reviewed here report short-term improvements in HSB, there is limited data on the long-term outcomes of these interventions. Future research should examine the long-term effectiveness of interventions, including monitoring progress over several years and assessing the impact of interventions on the autistic individual’s adaptive functioning and well-being.

### Identify Risk and Protective Factors

Further research is needed to identify factors that influence the risk of the development of HSBs in autistic adolescents that could inform the design of preventive interventions.

### Ethical Implications

Lastly, it is important to consider the ethical implications of treating HSBs in autistic adolescents, particularly when using medications that may have potential side effects or impact an individual’s rights or overall well-being. Future research should include thorough ethical considerations and informed consent processes to ensure the rights and well-being of participants are protected. Most studies identified in this REA did not report on ethics or consent processes when intervening and treating this cohort, including those with an intellectual disability or those with a reported diagnosis of “severe” autism.

Overall, the findings from our REA suggest that these research areas should be a focus for future studies to develop effective and tailored interventions for autistic adolescents with HSBs.

### Limitations

The “rapid” approach of the current review is not without its limitations. We chose to exclude certain types of research (unpublished) and included only peer-reviewed studies due to our knowledge of the limited work in this space. There, we did not search for study findings reported in conference papers and proceedings, dissertations, theses, or working papers, and we did not perform a random “dip” sample. As such, we could not determine whether the findings reported in these sources are notably different from those in studies published in peer-reviewed journals. Due to these limitations, this REA is prone to selection bias (Barends et al., 2017) and is an acknowledged limitation of the review.

## Conclusion

The limited, albeit growing, body of research on the management of HSBs in autistic adolescents highlights the complexity of this issue and the importance of a comprehensive approach to treatment. The studies we reviewed in this REA included CBT, pharmacological interventions, family involvement, and multidisciplinary teams. However, the research is generally of low quality and lacks the rigor required to make justifiable conclusions regarding what works in treating HSBs in autistic adolescents.

The available literature we reviewed has several limitations, including the scarcity of experimental designs, the absence of direct measures of change in HSBs, and the lack of follow-up data (Coskun & Mukaddes, 2008; Coskun et al., 2009). Moreover, most studies have focused on white male adolescents, which underscores the need for further research on HSBs in autistic females and other diversities. Future studies should address these limitations by employing more rigorous research designs, incorporating direct measures of HSBs, and conducting long-term follow-up assessments. Furthermore, research on prevention and intervention in the treatment of HSBs in autistic adolescents should expand to include diverse (but homogenous) populations, such as younger children and adults, individuals with varying levels of cognitive, social, and language abilities, and individuals from different cultural backgrounds. This will help to enhance our understanding of the prevalence, presentation, and management of HSBs in autistic people.

In conclusion, the management of HSBs in autistic adolescents is a critical area of abuse and trauma research and clinical practice. Although existing interventions have been evaluated, further research is needed to refine treatment approaches, establish the long-term effectiveness of interventions, and address the current gaps in the literature. Ultimately, a comprehensive and evidence-based approach to the treatment of HSBs in autistic individuals will contribute to improved outcomes and quality of life for affected individuals, their families, and the community.

**Table:** 

Critical Findings
The studies included in this review provided evidence for a range of interventions, including pharmacological treatments, cognitive-behavioral therapy, and other psychotherapeutic approaches. The findings, however, are to be interpreted with caution as the overall quality of the included studies was assessed to be low, with most studies rated at the lowest level for appropriateness and scored poorly in quality. Just one study (Visser et al., 2017), a randomized control trial, scored highly. We suggest that the literature is not robust enough to indicate a promising evidence-based approach for interventions for autistic adolescents who are at risk of or who display and engage in HSBs, and the findings are not transferable to practice. Additional research is required to better prepare healthcare professionals for addressing HSBs in autistic young people.
Implications
Different components (e.g., CBT, psychoeducation, pharmacological) in isolation and combination should be assessed, to determine the most effective treatment modalities for addressing HSBs in this group. Many of the studies reviewed in our REA utilized a multidisciplinary approach that included medication, behavioral therapy, and family therapy. Future research should continue to explore the effectiveness of individual and combined treatment modalities to develop comprehensive intervention packages for autistic adolescents with HSBs. Policymakers and healthcare organizations should support the development and dissemination of future resources, training programs, and evidence-based interventions to enhance the capacity of caregivers, educators, and clinicians to manage HSBs in autistic adolescents. Researchers should prioritize long-term follow-up studies, comparative effectiveness research, and the development of culturally and gender-responsive interventions to address the current gaps in the literature.

*Note.* CBT = cognitive-behavioral therapy; HSBs = harmful sexual behaviors; REA = rapid evidence assessment.

## Appendices

**Appendix A.** PICOC Table and Outline of Search Terms and Queries.

Population	Who?	Young people between the ages of 12 and 18 with an autism spectrum condition
Intervention	What or How?	To reduce the risk of this cohort engaging in HSBs or to reduce or eliminate the HSBs of the adolescent
Comparison	Compared to what?	NA
Outcome	What are you trying to accomplish/improve/change?	Acquire a good understanding of these kinds of interventions for this group and highlight gaps in research and current programs. This is so future work can build on these areas and help to further reduce the risk of HSBs in this group.
Context	In what kind of organization/circumstances?	Any organizational setting and context provides prevention, intervention, and treatment for HSBs in the population group as identified above.

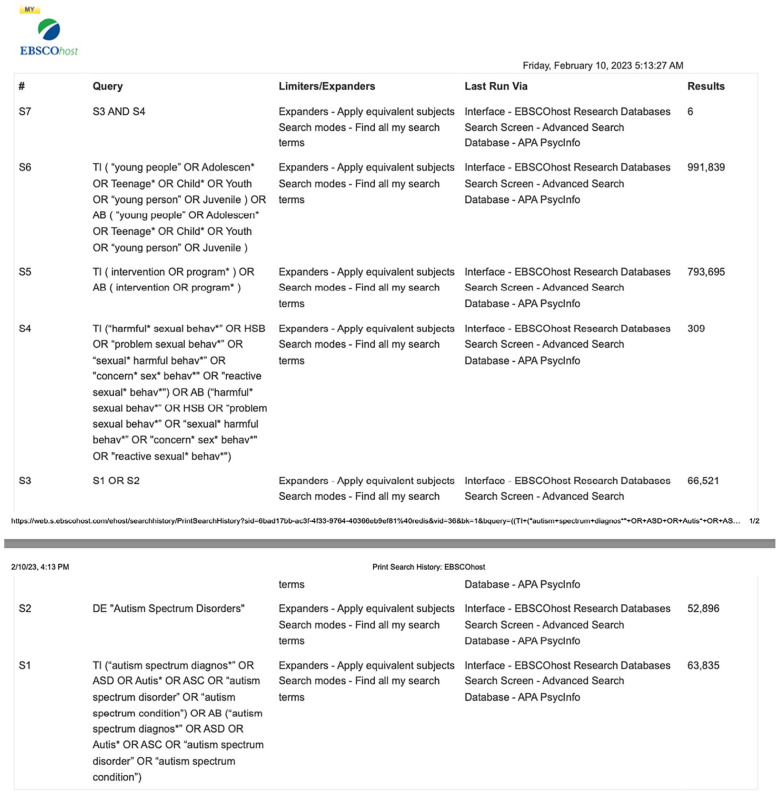

*Note.* HSBs = harmful sexual behaviors.

**Appendix B.** Search process. Limiters: None. Date search performed: February 10, 2023.

Databases	Concept 1	Concept 2	Concept 3	Results
Search terms TI/AB	adolescen* OR youth OR “young people” OR “young person” OR teen* OR child*	“harmful sexual* behavio*” OR “problem* sexual* behavio*” OR “concern* sexual* behavio*” OR “reactive sexual* behavio*” OR “sex* harm* behavio*” OR “sex* reactive behavio*”	“autism spectrum diagnos*” OR ASD OR autis* OR “autism spectrum disorder” OR ASC OR “autism spectrum condition”	
Medline MeSH	(MH “Adolescent”) OR (MH “Child”)	N/A	(MH “Autistic Disorder”) OR (MH “Autism Spectrum Disorder”)	2
PsycInfo	(ZG “adolescence (13–17 yrs)”)	N/A	DE “Autism Spectrum Disorders”	9
ERIC Thesaurus	(DE “Children”) OR (DE “Adolescents”)) OR (DE “Youth”)	N/A	DE “Autism” OR DE “Pervasive Developmental Disorders”	1
CINAHL Subject Terms	(MH “Child”) OR (MH “Adolescence”)	N/A	(MH “Autistic Disorder”)	5
Web of Science (all fields search)	Keywords as above	Keywords as above	Keywords as above	9
Scopus	Keywords as above	Keywords as above	Keywords as above	8
Total				34
Duplicates removed				16
To be screened				18
